# Time from submission to publication in urology journals: A look at publication times before and during Covid-19

**DOI:** 10.1016/j.heliyon.2023.e14233

**Published:** 2023-03-02

**Authors:** Netanja I. Harlianto, Zaneta N. Harlianto

**Affiliations:** a University Medical Center Utrecht and Utrecht University, Utrecht, the Netherlands; b Faculty of Medicine, Anton de Kom University of Suriname, Paramaribo, Suriname

**Keywords:** Urology, Bibliometric, Submission, Acceptance time, Publication times, Covid-19

## Abstract

**Background:**

The Covid-19 pandemic impacted scientific publishing, though it's effect on publication times in urology literature is unknown. The objective of our study were to determine and compare acceptance and publication times in general and specific urology journals, and to quantify these times before and during the Covid-19 pandemic.

**Methods:**

We identified all original articles published in seven urology journals in 2019 and 2021, and extracted data on submission, acceptance, online, and in-print publication times. Differences between groups were compared using Mann-Whitney U tests.

**Results:**

A total of 2880 articles were included, comprising 1601 articles published in 2021 and 1279 in 2019. Less experimental/animal studies were published in 2021 compared to 2019 (197 vs. 289). Time between submission and online publication was longer in 2021 (median 4.4 vs 3.3 months, p < 0.001), though acceptance times were not different (median 3.3 vs 3.3 months, p = 0.25). Prostate (median: 2.8 months, Neurourology and Urodynamics (median: 2.8 months) and Word Journal of Urology (median 2.9 months) had the shortest acceptance time in 2021. Time between submission and in-print publication ranged from 4.6 months (IQR: 3.6–6.8) for Prostate to 11.9 months (IQR: 9.8–13.2) for World Journal of Urology. Acceptance times were significantly longer in 2021 compared to 2019 for Prostate Cancer and Prostatic Diseases, Journal of Sexual Medicine, and Prostate. Moreover, time between submission and in print publication was longer in 2021 compared to 2019 for Journal of Sexual Medicine and Urologic Oncology: Seminars and Original Investigations, and shorter for Neurourology and Urodynamics. The median time to in print publication was lower for publications from US institutions (median 7.0 vs. 7.7 months in 2019 and 8.7 months vs 9.1 months in 2021).

**Conclusions:**

We identified journal specific acceptance and publication times and observed substantial differences between urology journals for the years 2019 and 2021.

## 1 Introduction

The dissemination of scientific knowledge can be a long and arduous process comprising the preparation, submission, and acceptance of research articles [1]. Timely publication of results is important in order to make results available for other researchers, patient care, and policymakers [2]. In recent years, more publishing models have emerged alongside traditional subscription-only articles, such as journals publishing fully open access or hybrid [3]. Some journals offer advanced online publication making uncorrected proofs promptly available as citable research items with a digital object identifier before typesetting has been done [4].

The Covid-19 pandemic had many effects on scientific publishing the past two years. More articles were submitted during the Covid-19 pandemic, which put a strain on the publishing system [5]. Moreover, Covid-19 related articles often benefited from accelerated review and (online) publication, which usually was at the expense of non-Covid-19 related articles for peer-reviewed journals in the field of infectious diseases [5].

The extent of submission and acceptance times may be an important journal parameter authors could take into account when selecting journals to submit their research to, alongside other factors such as the journal's impact factor and the fit of the research with the journal's aims and scope [6]. Publication times have been previously quantified for various fields, including ophthalmology and plastic surgery [7,8]. To our best knowledge, no previous study has investigated publication times between different journals in the field of urology. Furthermore, we wanted to quantify and compare publication times before and during the Covid-19 period. Therefore, the aim of the current bibliometric analysis was to report acceptance and publication times for original articles in the field of urology for the years 2019 and 2021.

## 2 Methods

### 2.1 Data sources

We reviewed all original articles published by seven urology journals with data available on submission and acceptance dates for issues published in 2019 and 2021 (each from January until December). The year 2020 was not included in our analysis given that for some journals with an extended time between submission and publication, the portion of articles submitted during the Covid-19 pandemic would be more easily skewed towards journals with a fast turnaround time regarding (online) publication. All articles published in the year 2021 were papers submitted during the Covid-19 pandemic. Journals were selected based on the Journal Citation Reports 2021, and included Prostate Cancer and Prostatic Diseases, World Journal of Urology, The Journal of Sexual Medicine, Urologic Oncology: Seminars and Original Investigations, Neurourology and Urodynamics, Prostate, and Journal of Pediatric Urology in order to include aspects of different fields within urology. Articles were excluded if they were supplements, case reports, editorials, technical notes, commentaries, letters to the editor, opinions, and non-systematic/narrative literature reviews. Narrative reviews were excluded because a no systematic review search is usually used in these types of articles. Moreover, a portion of journals commissioned and invited authors for narrative reviews, which could have an influence on publication times. Data were extracted on the country of the corresponding author, study design (randomized trial, prospective cohort, retrospective cohort, cross-sectional, case-control, case series, systematic review, database/population, and experimental/animal) and dates on submission, acceptance, online, and in-print publication. We calculated the time between submission and acceptance; time between acceptance and online publication; time between acceptance and in print publication; and time between submission and in print publication.

### 2.2 Statistical analysis

Continuous data were presented as median and interquartile range (IQR), and categorical data as frequencies and percentages. Differences between groups on study design, country, and articles on Covid-19 were analyzed using the Mann-Whitney *U* test and the Kruskal-Wallis test for nonparametric data. To evaluate the times from submission to acceptance and publication, we utilized Kaplan-Meier analyses. Statistical analysis was performed using R, version 3.6.3 (R Foundation for Statistical Computing, Vienna, Austria). Statistical significance was set at a p-value < 0.05.

## 3 Results

### 3.1 Characteristics of included journals

A total of 2880 articles were included in the current study, of which 1601 were published in 2021 and 1279 in 2019. Prostate Cancer and Prostatic Diseases was the journal with the highest impact factor (5.455), whereas the Journal of Pediatric Urology had the lowest impact factor (1.921). Most journals published monthly issues (including World Journal of Urology, The Journal of Sexual Medicine, Urologic Oncology: Seminars and Original Investigations, and Prostate) and Prostate Cancer and Prostatic Diseases published the least issues with four issues per year. Prostate Cancer and Prostatic Diseases, World Journal of Urology, Urologic Oncology: Seminars and Original Investigations, and Journal of Pediatric Urology published more articles in 2021 compared to 2019. The most original articles were published by World Journal of Urology (Table 1). In 2021, the Journal of Sexual Medicine, Prostate, and Prostate Cancer and Prostatic Diseases published 13 articles that were rapid communications of short novel findings, of which 1 (7.7%) article was related to Covid-19.

**Table 1:** Journal characteristics.

Journal	Year	Impact Factor	No. of Issues	No. of original articles, N (%)	Acceptance Time in months median (IQR)	Online Publication in months median (IQR)	Acceptance to In Print Pubblication in months median (IQR)	Total Time in months median (IQR)
Prostate Cancer and Prostatic Disease	2021	5.455	4	110	3.4 (2.7–4.2)*	0.8 (0.6–1.2)*	7.5 (6.8–8.4)*	11.1 (10.1–12.5)
	2019	4.311	4	62	2.1 (1.7–3.2)*	2.0 (1.4–2.8)*	8.2 (7.4–9.9)*	10.8 (9.5–12.1)
World Journal of Urology	2021	3.661	12	462	2.9 (2.1–3.9)	0.6 (0.4–0.9)*	9.2 (6.7–10.0)*	11.9 (9.8–13.2)
	2019	3.217	12	249	3.1 (2.1–4.2)	0.4 (0.3–0.5)*	8.7 (8.2–9.1)*	11.7 (10.5–12.9)
The Journal of Sexual Medicine	2021	3.937	12	175	4.4 (3.4–5.7)*	2.0 (1.6–2.5)*	2.4 (2.1–2.7)	6.8 (5.8–8.4)*
	2019	3.293	12	179	3.7 (2.8–5.4)*	1.8 (1.5–1.9)*	2.4 (2.1–2.8)	6.1 (5.0–7.7)*
Neurourology and Urodynamics	2021	2.367	8	197	2.8 (1.9–4.0)	1.0 (0.7–1.2)*	2.5 (2.0–3.0)*	5.5 (4.2–6.7)*
	2019	2.037	8	237	2.8 (2.0–4.1)	1.4 (1.0–2.0)*	3.1 (2.4–3.7)*	6.2 (5.1–7.7)*
Prostate	2021	4.012	16	136	2.8 (2.0–4.9)*	0.8 (0.6–1.1)*	1.6 (1.3–2.1)*	4.6 (3.6–6.8)
	2019	3.279	16	179	2.1 (1.3–3.4)*	1.2 (1.0–1.6)*	2.3 (1.8–2.7)*	4.7 (3.8–5.9)
Urologic Oncology: Seminars and Original Investigations	2021	2.954	12	281	3.3 (2.5–4.5)*	1.4 (1.0–2.0)*	5.4 (3.6–6.9)*	9.4 (7.9–10.3)*
	2019	2.882	12	189	3.8 (2.9–5.2)*	1.1 (0.9–1.8)*	3.2 (2.3–3.9)*	7.3 (6.2–8.3)*
Journal of Pediatric Urology	2021	1.921	6	240	4.5 (2.9–6.4)	0.3 (0.2–0.4)*	3.6 (2.8–4.2)	8.2 (6.4–9.6)
	2019	1.578	6	184	4.7 (3.4–6.8)	0.3 (0.3–0.5)*	3.3 (2.5–4.1)	8.5 (6.6–10.4)

IQR: interquartile range; * Mann-Whitney-U p-value < 0.05 between 2021 and 2019.

### 3.2 Acceptance and publication times

**Fig. 1:**
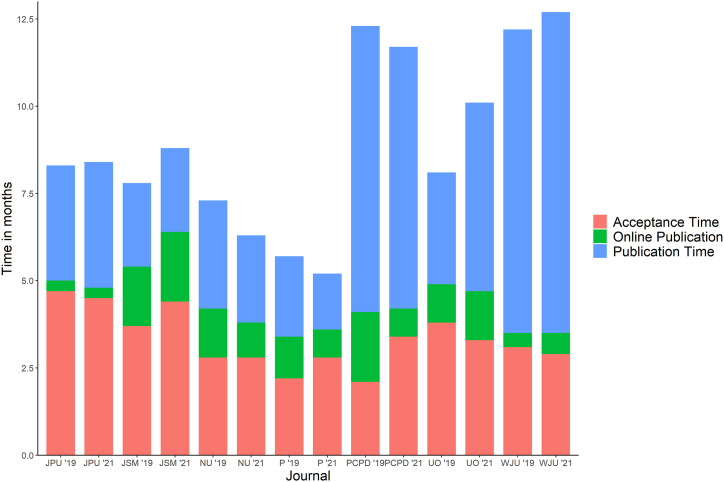
Acceptance and publication times by year for each journal. JPU: Journal of Pediatric Urology; PCPD: Prostate Cancer and Prostatic Diseases; WJU: World Journal of Urology; JSM: The Journal of Sexual Medicine; UO: Urologic Oncology: Seminars and Original Investigations; NU: Neurourology and Urodynamics, P: Prostate.

**Fig. 2:**
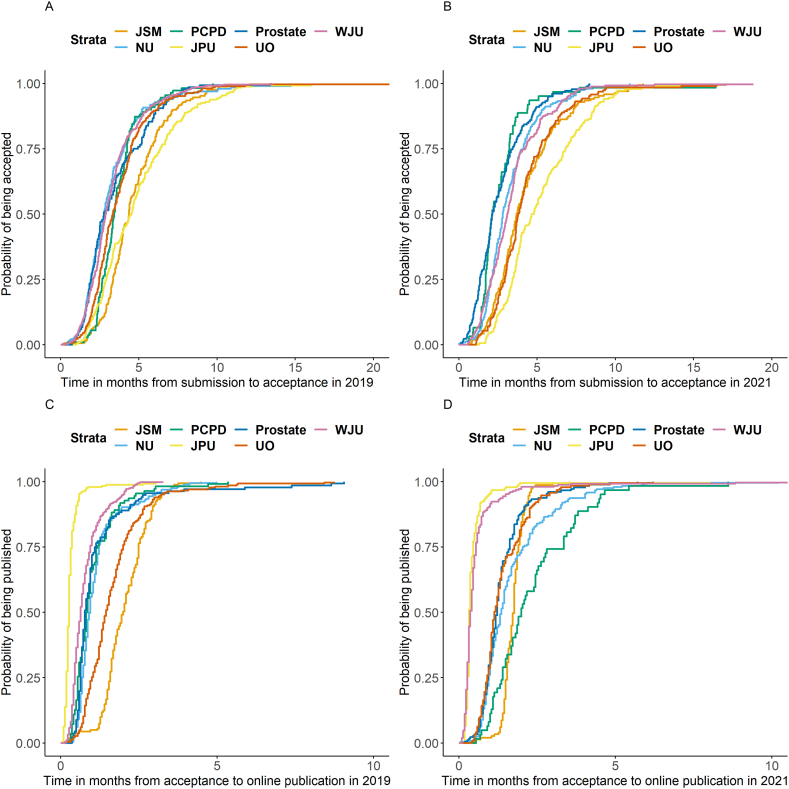
A: Time between submission and acceptance in 2019; B: Time between submission and acceptance in 2021; C: Time between acceptance and online publication in 2019; D: Time between acceptance and online publication in 2021. JPU: Journal of Pediatric Urology; PCPD: Prostate Cancer and Prostatic Diseases; WJU: World Journal of Urology; JSM: The Journal of Sexual Medicine; UO: Urologic Oncology: Seminars and Original Investigations; NU: Neurourology and Urodynamics.

**Fig. 3:**
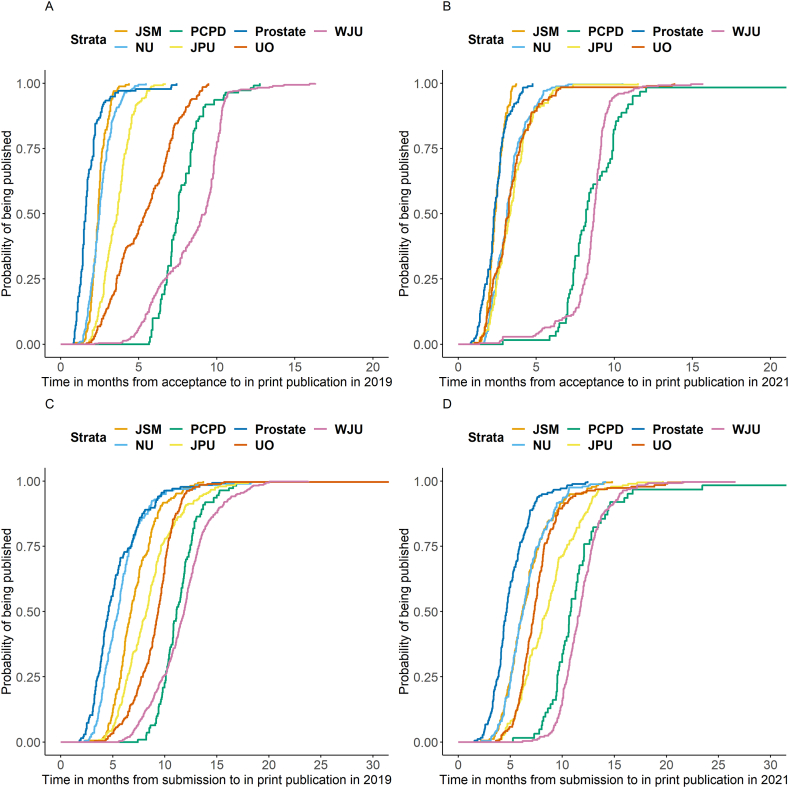
A. Time between acceptance and in print publication in 2019; B: Time between acceptance and in print publication in 2021; C: Time between submission and in print publication in 2019; D: Time between submission and in print publication in 2021. JPU: Journal of Pediatric Urology; PCPD: Prostate Cancer and Prostatic Diseases; WJU: World Journal of Urology; JSM: The Journal of Sexual Medicine; UO: Urologic Oncology: Seminars and Original Investigations; NU: Neurourology and Urodynamics.

Overall, the total time between submission and in print publication was longer in 2021 compared to 2019 (median 9.0 vs 7.5 months, p < 0.001) and a longer time between acceptance and in print publication was seen in 2021 (median 4.4 vs 3.3 months, p < 0.001). The time between submission and acceptance did not differ between 2019 and 2021 (median 3.3 vs. 3.3 months, p = 0.25). Prostate Cancer and Prostatic Diseases (median: 2.1 months; IQR: 1.7–3.2) and Prostate (median: 2.1 months; IQR: 1.3–3.4) had the shortest time between submission and acceptance in 2019 (Table 1). In 2021, Prostate (median: 2.8 months; IQR: 2.0–4.9), Neurourology and Urodynamics (median: 2.8 months; IQR: 1.9–4.0) and Word Journal of Urology (median 2.9 months; IQR: 2.1–3.9) had the shortest time between submission and acceptance of all seven journals (Fig. 1). In 2019, the Journal of Pediatric Urology (median 0.3 months; IQR: 0.3–0.5) and World Journal of Urology (median 0.4 months; IQR: 0.3–0.5) had the shortest time between acceptance and online publication, whereas the Journal of Sexual Medicine (median 1.7 months; IQR: 1.5–1.9) and Prostate Cancer and Prostatic Diseases (median 2.0 months; IQR: 1.4–2.8) had the longest time between acceptance and online publication. Journals with the longest time between submission and in-print publication were World Journal of Urology and Prostate Cancer and Prostatic Diseases. Kaplan Meier analyses for 2021 and 2019 are shown in Figs. 2A–D and 3A–D, respectively.

When comparing acceptance and publication times for each journal between 2019 and 2021, time between submission and acceptance was significantly longer for Prostate Cancer and Prostatic Diseases, Journal of Sexual Medicine, and Prostate, but not for other journals. Urologic Oncology: Seminars and Original Investigations had significantly lower time between submission and acceptance in 2021. Significantly longer time between acceptance and in print publication were observed in 2021 compared to 2019 for World Journal of Urology and Urologic Oncology: Seminars and Original Investigations, which was lower in 2021 for Prostate Cancer and Prostatic Diseases, Neurourology and Urodynamics, and Prostate.

Time between submission and in print publication was longer in 2021 for Journal of Sexual Medicine and Urologic Oncology: Seminars and Original Investigations, shorter for Neurourology and Urodynamics, whereas time between submission and in print publication was not different for the remainder of journals (Table 1).

### 3.3 Acceptance times by study design and country

In 2021, most studies were retrospective cohort studies (n = 433, 26.5%) and cross-sectional studies (n = 394, 24.6%), whereas in 2019 most studies were cross-sectional (n = 356, 27.8%) and animal/experimental (n = 289, 22.6%). In 2021, animal/experimental studies accounted for 12.3% (n = 197) of all publications. Acceptance and publication times for 2021 and 2019 by study design are listed in Supplementary Table S1.

In both 2021 and 2019 the majority of publications were from US institutions, 30.2% and 32.3%, respectively. The median time between submission and in print publication was lower for publications from US institutions (median 7.0 vs. 7.7 months in 2019 and 8.7 months vs 9.1 months in 2021), compared to publications from other institutions (Supplementary Table S2). US authors published less articles compared to other countries in World Journal of Urology and Prostatic Cancer and Prostatic Diseases in both 2019 (19.8% vs. 24.3%) and 2021 (24.6% vs. 35.7%). A total of 3 2 articles were published in 2021 with “Covid-19”, “Corona”, or “Sars” in the title. These articles did not differ in time between acceptance and in print publication and time between submission and in print publication compared to non-Covid articles, though a longer time between submission and acceptance was observed for Covid-19 articles (median 3.9 vs 3.3 months, p = 0.04) (Supplementary Table S2).

## 4 Discussion

As there is a scarcity of data on studies reporting publication times in urology, we aimed to quantify acceptance and publication times of original articles using a bibliometric analytical approach for the years 2019 and 2021, in order to quantify the effects of Covid-19 on publication and acceptance times. We found that in 2021, in general, time between submission and in print publication was longer compared to 2019 (median 4.4 vs 3.3 months), though interestingly, times from submission and acceptance on average was not different between years (median 3.3 vs 3.3 months). Furthermore, we identified journal-specific differences in acceptance and online and in print publication times. The extended time to in-print publication could be due to the accumulated backlog of articles waiting to be processed by the journal publisher, given the rise in articles in 2021. Contrary to our expectations, not all journals had prolonged time between submission and acceptance during the Covid-19 period, as no differences were seen for World Journal of Urology, Journal of Pediatric Urology, and Neurourology and Urodynamics, and Urologic Oncology: Seminars and Original Investigations even had a shorter time between submission and acceptance in 2021 compared to 2019. These differences were also observed for time between acceptance and in print publication, and time between submission and in print publication, as most journals had either longer or shorter time between acceptance and in print publication, and time between submission and in print publication between years, instead of similar time between acceptance and in print publication, and time between submission and in print publication between 2019 and 2021. Overall, more articles were published in 2021 compared to 2019, although only four of the seven included journals published more original articles in 2021. This could also be partly influenced by the increase in publications over time. It was apparent that fewer original experimental and animal studies were published in 2021, despite the overall number of original articles being higher in 2021. This decrease in animal and experimental studies could be attributed to the worldwide lockdowns of research centers and universities during the Covid-19 pandemic to reduce the spread of infections [9]. Moreover, it is thought that onsite delays of clinical trials resulted in less overall trials published as patient recruitment was disrupted [10], though we did not observe less trials published in 2021 compared to 2019. In our study only 32 (1.9%) articles related to Covid-19 published in 2021 were identified, which had no different time between acceptance and in print publication, and time between submission and in print publication compared to non-Covid-19 articles. This may be explained by the relatively low number of Covid-19 related articles which weakened the statistical effect, though only 1 paper related to Covid-19 was identified as a rapid communication, which we were not able to further assess. The rise of Covid-19 articles may be more apparent in infectious disease journals and other fields [11], whereas fewer Covid-19 related research was performed in the field of urology. Other Covid-19 articles could have been reported in other high impact urology journals, which we did not include as they did not report publication times. In our results, articles published by US institutions had longer time to in print publication, though there was no clear relation for acceptance times and time to online publication for each year. This extended time to in print publication may be explained because authors from US institutions published less articles in World Journal of Urology and Prostate Cancer and Prostatic Diseases, journals with the longest time to in print publication.

We studied the acceptance and publication times in relation to various study specific factors such as study design and the country of the corresponding author, though we could not quantify other “hidden” contributors affecting acceptance and publication times, which was largely outside the scope of this study. A recent systematic review encompassing more than 30 studies identified that publication times in the literature are substantially different, ranging from one month to almost two years. This imbalance was also observed for acceptance times, as these averages ranged from 1.64 months to nine months [12].

Various factors can be attributed to publication and acceptance times. Authors are responsible for revising their manuscript based on the reviewer's comments. Furthermore, journal selection should be based on the fit and merit of the paper, instead of the journal impact factor, to limit future resubmissions in the case of a rejection [13].

Though, acceptance times are predominantly dependent on journal factors, editorial handling times, more specifically for finding suitable peer reviewers, assessing reviewer comments, and if applicable, editorial board manuscript discussions [14]. High quality peer review requires the selection of the right reviewers and finding peer reviewers in a timely manner is important for ensuring that submitted articles do not experience publication delays. It is estimated that reviewers take on average 4–5 h per review and that approximately 20% of reviewers are responsible for 69–94% of the reviews [14,15].

Time to acceptance of an article may be affected by the time it takes for reviewers to start on the review. Reviewers are usually given four weeks to complete a peer review, though it can be challenging to allocate time to review within their existing busy schedule, next to their own research duties, or patient care.

Authors are more likely to engage in peer review if the topic aligns with their own research. The most common reason for declining reviews according to surveyed authors was a lack of time [16]. Time spent peer-reviewing could be alternatively used for other research related activities [13]. Incentives for participating in peer review exist to encourage peer review, such as limited journal subscriptions, article processing fee discounts, or reviewer acknowledgements on journal websites or Web of Science and Publons [16,17].

The limitations of our study warrant discussion. First, our results may have been affected by the number of original articles each journal publishes yearly and selection bias could be present given that not all journals had acceptance times available. Second, peer review times are affected by various factors related to editor handling times, as well as authors resubmission [13], which we were not able to quantify or assess. It is also thought that journals with higher impact factors, on average, receive more submissions on a yearly basis, which has an impact on peer review times [18]. Finally, the limited amount of Covid-19 articles published in 2021 could skew and affect acceptance and publication times for this subgroup. Ideally, a longitudinal analysis of each year would be conducted for each journal, particularly of the 2022 issues.

## 5 Conclusion

In this first bibliometric study in the field of urology, we found substantial differences in acceptance and publication times for general and field-specific urology journals. Time from submission to in print publication was longer in 2021 with 4.5 months compared to 3.3 months in 2019, with some variation among journals. Acceptance times were on average 3.3 months and were not different between 2019 and 2021.

## Author contribution statement

Netanja I Harlianto; Zaneta N Harlianto: Conceived and designed the experiments; Performed the experiments; Analyzed and interpreted the data; Contributed reagents, materials, analysis tools or data; Wrote the paper.

## Funding statement

This research did not receive any specific grant from funding agencies in the public, commercial, or not-for-profit sectors.

## Data availability statement

Data will be made available on request.

## Declaration of interest's statement

The authors declare that they have no known competing financial interests or personal relationships that could have appeared to influence the work reported in this paper.
