# Supplementary material for: Time from submission to publication in urology journals: A look at publication times before and during Covid-19
Source: Heliyon. 2023 Mar 2;9(3):e14233. doi: 10.1016/j.heliyon.2023.e14233 (PMC10009718; doi:10.1016/j.heliyon.2023.e14233)
Supplement: Multimedia component 1 [file mmc1.docx]

**Title:** Time from submission to publication in urology journals: a look at publication times before and during Covid-19
**Authors:** Netanja I Harlianto & Zaneta N. Harlianto**;
Email address corresponding author**: N.I.Harlianto@umcutrecht.nl

**Supplementary Table S1. Acceptance and publication times by year and study design**

| **2021** | **N (%)** | **Acceptance Time in months  median (IQR)** | **Online Publication in months  median (IQR)** | **Acceptance to in print publicationT in months median (IQR)** | **Total Time in months median (IQR)** |
| --- | --- | --- | --- | --- | --- |
| Systematic review | 130 (8.1) | 3.1 (2.2 – 4.2) | 1.0 (0.5 – 1.7) | 4.7 (3.0 – 7.20 | 8.4 (6.7 – 10.7) |
| Randomized Trial | 60 (3.7) | 3.0 (2.0 – 3.9) | 0.8 (0.5 – 1.6) | 5.4 (2.9 – 8.7) | 8.1 (5.8 – 11.8) |
| Prospective cohort | 180(11.2) | 3.2 (2.4 – 4.9) | 0.8 (0.4 – 1.3) | 5.3 (2.6 – 8.3) | 9.0 (6.8 – 11.6) |
| Retrospective cohort | 435 (26.5) | 3.1 (2.3 – 4.3) | 0.8 (0.4 – 1.3) | 5.5 (2.9 – 8.1) | 9.2 (6.5 – 11.6) |
| Cross-sectional | 394 (24.6) | 3.7 (2.5 – 5.1) | 0.8 (0.5 – 1.5) | 3.7 (2.4 – 7.6) | 9.0 (6.4 – 11.6) |
| Case-control | 36 (2.2) | 3.6 (2.7 – 4.8) | 1.0 (0.4 – 1.8) | 3.6 (2.6 – 6.0) | 8.0 (6.4 – 11.0 |
| Population/database | 141 (8.8) | 3.3 (2.4 – 4.4) | 1.0 (0.5 – 1.8) | 4.8 (2.7 – 7.5) | 8.8 (6.7 – 10.9) |
| Animal/Experimental | 197 (12.3) | 3.5 (2.4 – 5.4) | 0.9 (0.6 – 1.5) | 3.3 (2.2 – 6.8) | 8.1 (5.8 – 11.1) |
| Case-series | 18 (1.1) | 4.4 (3.2 – 7.9) | 0.4 (0.3 – 1.2) | 3.1 (2.5 – 4.2) | 8.3 (6.4 – 13.1) |
| Other | 10 (0.6) | 3.5 (3.0 – 4.1) | 1.2 (0.9 – 2.2) | 6.3 (4.1 – 7.0) | 9.8 (9.0 – 10.1) |
| **2019** | **N (%)** | **AT in months  median (IQR)** | **OP in months  median (IQR)** | **PT in months median (IQR)** | **TT in months median (IQR)** |
| Systematic review | 84 (6.6) | 3.0 (2.0 – 4.9) | 1.2 (0.5 – 1.9) | 3.4 (2.6 – 6.1) | 7.6 (6.0 – 9.5) |
| Randomized Trial | 48 (3.8) | 3.1 (2.6 – 4.2) | 1.2 (0.6 – 1.7) | 3.5 (2.4 – 5.1) | 6.8 (5.7 – 10.6) |
| Prospective cohort | 106 (8.3) | 3.2 (2.2 – 3.9) | 1.1 (0.4 – 1.9) | 4.8 (3.0 – 8.5) | 9.6 (6.1 – 11.9) |
| Retrospective cohort | 215 (16.8) | 3.5 (2.4 – 4.8) | 0.9 (0.4 – 1.4) | 3.9 (2.7 – 8.4) | 8.6 (6.3 – 11.2) |
| Cross-sectional | 356 (27.8) | 3.7 (2.5 – 5.5) | 1.0 (0.4 – 1.7) | 3.1 (2.4 – 4.9) | 8.1 (6.0 – 10.6) |
| Case-control | 29 (2.3) | 3.7 (3.2 – 5.4) | 1.0 (0.4 – 1.8) | 2.8 (2.3 – 4.3) | 7.7 (6.1 – 10.4) |
| Population/database | 99 (7.7) | 3.1 (1.9 – 4.1) | 1.1 (0.5 – 1.5) | 3.7 (2.8 – 7.8) | 8.2 (6.5 – 10.6) |
| Animal/Experimental | 289 (22.6) | 2.7 (1.8 – 4.0) | 1.2 (0.8 – 1.7) | 2.6 (2.1 – 3.6) | 5.7 (4.4 – 7.2) |
| Case-series | 38 (3.0) | 4.0 (3.2 – 6.4) | 0.4 (0.3 – 1.5) | 3.5 (2.4 – 7.3) | 9.4 (7.2 – 11.9) |
| Other | 15 (1.2) | 2.8 (2.3 – 4.3) | 1.0 (0.6 – 1.7) | 3.4 (2.4 – 6.4) | 7.3 (5.6 – 11.6) |

IQR: interquartile range;

**Supplementary Table S2.** **Acceptance and publication times by year and author affiliation**

| **2021** | **N (%)** | **Acceptance Time in months  median (IQR)** | **Online Publication in months  median (IQR)** | **PT in months median (IQR)** | **TT in months median (IQR)** |
| --- | --- | --- | --- | --- | --- |
| **COVID-19** |  |  |  |  |  |
| Yes | 32 | 3.9 (2.9 – 5.5)* | 1.0 (0.4 – 1.6) | 3.7 (2.4 – 5.5) | 8.5(6.4 – 10.1) |
| No | 1569 | 3.3 (2.4 – 4.7)* | 0.8 (0.5 – 1.4) | 4.4 (2.5 – 7.7) | 9.0 (6.3 – 11.4) |
| **Institution** |  |  |  |  |  |
| US institution | 484 (30.2) | 3.3 (2.4 – 4.8) | 0.9 (0.5 – 1.6) | 3.9 (2.4 – 6.9)* | 8.7 (6.0 – 10.8)* |
| Other | 1117 (69.8) | 3.3 (2.4 – 4.7) | 0.8 (0.5 – 1.4) | 4.7 (2.6 – 8.2)* | 9.1 (6.5 – 11.6)* |
| **2019** | **N (%)** | **AT in months  median (IQR)** | **OP in months  median (IQR)** | **PT in months median (IQR)** | **TT in months median (IQR)** |
| **Institution** |  |  |  |  |  |
| US institution | 414 (32.3) | 3.2 (2.1 – 4.4)* | 1.1 (0.6 – 1.8)* | 3.1 (2.2 – 4.5) | 7.0 (5.2 – 9.7)* |
| Other | 865 (67.7) | 3.4 (2.3 – 4.8)* | 1.0 (0.5 – 1.6)* | 3.3 (2.5 – 6.4) | 7.7 (5.8 – 10.7)* |

IQR: interquartile range; * Mann-Whitney-U p-value <0.05
